# A Comprehensive Metabolism-Related Gene Signature Predicts the Survival of Patients with Acute Myeloid Leukemia

**DOI:** 10.3390/genes15010063

**Published:** 2023-12-31

**Authors:** Yujia Zhai, Heng Shen, Hui Wei

**Affiliations:** State Key Laboratory of Experimental Hematology, National Clinical Research Center for Blood Diseases, Haihe Laboratory of Cell Ecosystem, Institute of Hematology & Blood Diseases Hospital, Chinese Academy of Medical Sciences & Peking Union Medical College, Tianjin 300020, China; zhaiyujia@ihcams.ac.cn (Y.Z.); shenheng@ihcams.ac.cn (H.S.)

**Keywords:** acute myeloid leukemia, metabolism-related gene, prognostic model, survival analysis, RNA-seq analysis

## Abstract

(1) Background: Acute myeloid leukemia (AML) is a clonal malignancy with heterogeneity in genomics and clinical outcome. Metabolism reprogramming has been increasingly recognized to play an important role in the leukemogenesis and prognosis in AML. A comprehensive prognostic model based on metabolism signatures has not yet been developed. (2) Methods: We applied Cox regression analysis and the least absolute shrinkage and selection operator (LASSO) normalization to establish a metabolism-related prognostic gene signature based on glycolysis, fatty acid metabolism, and the tricarboxylic acid cycle gene signatures. The Cancer Genome Atlas-Acute Myeloid Leukemia-like (TCGA-LAML) cohort was set as the training dataset for model construction. Three independent AML cohorts (GSE37642, GSE10358, and GSE12417) combined from Gene Expression Omnibus (GEO) datasets and the Beat-AML dataset were retrieved as two validation sets to test the robustness of the model. The transcriptome data and clinic information of the cohorts were enrolled for the analysis. (3) Results: Divided by the median value of the metabolism risk score, the five-year overall survival (OS) of the high-risk and low-risk groups in the training set were 8.2% and 41.3% (*p* < 0.001), respectively. The five-year OS of the high-risk and low-risk groups in the combined GEO cohort were 25.5% and 37.3% (*p* = 0.002), respectively. In the Beat-AML cohort, the three-year OS of the high-risk and low-risk groups were 16.2% and 40.2% (*p* = 0.0035), respectively. The metabolism risk score showed a significantly negative association with the long-term survival of AML. Furthermore, this metabolism risk score was an independent unfavorable factor for OS by univariate analysis and multivariate analysis. (4) Conclusions: Our study constructed a comprehensive metabolism-related signature with twelve metabolism-related genes for the risk stratification and outcome prediction of AML. This novel signature might contribute to a better use of metabolism reprogramming factors as prognostic markers and provide novel insights into potential metabolism targets for AML treatment.

## 1. Introduction

Acute myeloid leukemia (AML) is a malignant clonal disease characterized by a blockade in the differentiation of hematopoietic stem and progenitor cells, leading to the abnormal proliferation of immature myeloblasts. Despite advancements in hematopoietic stem cell transplantation (HSCT) and novel agents, the prognosis of AML patients remains suboptimal, with approximately 70% of patients who achieve remission eventually experiencing relapse. The 5-year overall survival (OS) rate is still unsatisfactory [1]. Risk stratification based on cytogenetics and genomic signatures has been widely used in clinical practice to identify favorable, intermediate, and unfavorable risk groups. However, due to the genetic mutation diversity and high heterogeneity of AML, current risk stratification methods have limitations in accurately predicting the outcome of all patients, particularly those with multiple mutations. Therefore, there is an urgent need to identify prognostic features that can serve as novel therapeutic targets and be applied in risk stratification and treatment guidance to improve clinical outcomes.

Metabolism reprogramming has gained increasing recognition for its significant role in tumor cell proliferation, invasion, and survival. This recognition has opened up promising avenues for the development of novel therapeutic targets. Previous studies have demonstrated that cancer cells, unlike normal cells, preferentially utilize aerobic glycolysis and enhance flux through the truncated tricarboxylic acid (TCA) cycle to support tumor growth. In vitro results have shown that increased glycolysis contributes to AML cell resistance to apoptosis induction by chemotherapeutics [2]. Conversely, the inhibition of glycolysis suppresses leukemia cell proliferation and enhances the cytotoxicity of cytarabine [3]. Specific mutant isocitrate dehydrogenase 1 and 2 (IDH1/2) inhibitors reduce the catalyzation of mutant IDH1/2, which converts α-ketoglutarate (α-KG) in the TCA cycle to the oncometabolite 2-hydroxyglutarate (2-HG). This alteration competes with α-KG and affects DNA and histone demethylases, ultimately promoting leukemogenesis [4,5]. Moreover, disrupting the TCA cycle in primary AML blasts using BCL-2 inhibitors combined with hypomethylating agents (HMAs) has been shown to eliminate leukemia stem cells (LSCs) by suppressing oxidative phosphorylation (OXPHOS) [6]. Additionally, AML cells rely more on fatty acid β-oxidation for energy production and membrane biogenesis, in contrast to the repressed de novo synthesis of fatty acids in differentiated cells [7]. Several studies have demonstrated that different rate-limiting enzymes in fatty acid oxidation and synthesis are overexpressed in certain AML cell lines and are associated with worse patient survival [8]. A phase 2 clinical trial showed that the combination of statins and chemotherapy improved the complete remission (CR) and CR with incomplete count recovery (CRi) rates in relapsed/refractory (R/R) AML [9].

Based on the aforementioned studies, we conducted a study to establish a metabolism-related prognostic model that mainly focuses on the combination of genes related to glycolysis, fatty acid metabolism, and the TCA cycle for predicting long-term prognosis in AML patients. We obtained gene expression profiles and corresponding clinical information of patients from the Cancer Genome Atlas-Acute Myeloid Leukemia-like (TCGA-LAML) project to perform Cox regression analysis and least absolute shrinkage and selection operator (LASSO) normalization. Our findings revealed the prognostic value of metabolism signatures and provided novel insights into potential metabolism-related therapeutic targets for AML.

## 2. Materials and Methods

### 2.1. Data Sources and Patient Characteristics

The RNA-seq data of bone marrow (BM) samples and clinical information of 131 adult AML patients in the TCGA-LAML project [10] were extracted from the GDC Data Portal site (https://portal.gdc.cancer.gov/, accessed on 1 October 2022) as a training set, after filtering out patients without treatment or diagnosed as AML-M3. The TCGA-LAML database was described in detail in https://gdc.cancer.gov/about-data/publications/laml_2012 (accessed on 1 October 2022). Due to the distinct molecular mechanisms, unique treatment approaches, and generally more favorable prognosis associated with AML-M3 compared to other AML subtypes, patients with AML-M3 were excluded from this study. Three independent AML cohorts (GSE37642 [11], GSE10358 [12], and GSE12417 [13]) in combination with Gene Expression Omnibus (GEO) datasets and the Beat-AML dataset [14] from the VIZOME website (http://www.vizome.org/aml/, accessed on 1 October 2022) were retrieved as two validation sets. GSE37642, GSE10358, and GSE12417 were described in https://www.ncbi.nlm.nih.gov/geo/query/acc.cgi?acc=GSE37642 (accessed on 1 October 2022), https://www.ncbi.nlm.nih.gov/geo/query/acc.cgi?acc=GSE10358 (accessed on 1 October 2022) and https://www.ncbi.nlm.nih.gov/geo/query/acc.cgi?acc=GSE12417 (accessed on 1 October 2022), respectively. The Beat-AML database was described in https://www.cancer.gov/ccg/blog/2019/beataml (accessed on 1 October 2022). After excluding patients without RNA-seq data or without survival information and removing batch effects by sva packages (version 3.48.0) in R [15], ultimately, 300 and 252 AML patients were included for validation analysis, respectively. The overall features of cases in each database enrolled in our study are demonstrated in Table S1.

The biological and clinical data referring to patients of the three cohorts are summarized in Table 1. The follow-up duration of Beat-AML was significantly shorter than the other two cohorts (*p* < 0.001). Differences in general clinical information including median age, gender, WBC count at diagnosis, and BM blast of training and validation sets are not statistically significant. There were significantly fewer AML-M1 and AML-M2 patients in the Beat-AML cohort than the other two cohorts (*p* < 0.001), and more favorable risk cases in the Beat-AML than in the TCGA-LAML cohort (*p* < 0.001). Most patients in the TCGA and Beat-AML cohorts were treated with “7 + 3” standard chemotherapy. Patients in the GEO datasets were treated according to the AMLCG-1999 (NCT00266136) protocol, including daunorubicin and high-dose cytarabine in induction, and the CALGB (NCT00002925) protocol, including cytarabine, daunorubicin, and etoposide.

The “cytogenetic risk” referred to 2022 ELN (European Leukemia Net) risk classification by genetics at initial diagnosis [16]. Common favorable cytogenetic abnormalities included t(8;21), inv(16), and *NPM1* mutations without *FLT3*-internal tandem duplications (*ITD*) mutations. Intermediate cytogenetic abnormalities included *FLT3-ITD* mutations, t(9;11), and cytogenetic features that do not belong to favorable or high-risk groups. High-risk cytogenetic abnormalities mainly include t(v;11) complex karyotypes and *TP53* mutations. Notably, there is necessary information for determining cytogenetic risk in the TCGA-LAML and Beat-AML cohorts to stratify these cases, while there is not enough information for stratification in GEO cohorts.

### 2.2. Differentiation of Metabolic Status of the Patients in the TCGA-LAML Dataset

The upregulation of metabolism-related pathways is known to be associated with increased metabolic activity, and the key enzymes involved in metabolism play a critical role in determining the rate of metabolic processes. Rate-limiting enzymes can affect the overall speed of the entire metabolic pathway. Table 2 shows the key rate-limiting enzymes in glycolysis, the TCA cycle, and fatty acid metabolism, and their corresponding gene symbols.

While the relationship between enzyme activity and metabolic flux is complex, several studies have demonstrated that gene expression levels can partially reflect metabolic activity [17,18]. The expression value of each enzyme was the average of the sum expression value of all gene types encoding this enzyme. Firstly, we used the average of the sum of HK1, HK2, and HK3 expression values to represent the expression value of hexokinase. This way, the expression value of phosphofructokinase-1 and pyruvate kinase were calculated. Secondly, the sum of the expression values of these three rate-limiting enzymes in glycolysis was calculated to represent the glycolysis pathway activity. Similarly, we calculated the sum of the expression values of corresponding rate-limiting enzymes of the TCA cycle pathway and fatty acid metabolism pathway. Finally, we add them together to distinguish the metabolic status of patients. By the median value of the total expression value of seven key rate-limiting enzymes of the three metabolism pathways, we separated the TCGA-LAML training cohort into metabolism ^high^ and metabolism ^low^ groups.

### 2.3. Gene Set Enrichment Analysis

Gene set enrichment analysis (GSEA) is a bioinformatics method for analyzing large-scale gene expression data, which was accomplished through the GSEA software v4.3.2 (Broad Institute) [19]. It aims to investigate the relationship between gene sets and specific biological conditions. To access annotated gene sets for GSEA, we utilized the Molecular Signature Database (MSigDB), which is a comprehensive resource of annotated gene sets for use in GSEA software, available at https://www.gsea-msigdb.org/gsea/msigdb/index.jsp (accessed on 1 October 2022). We obtained the c2.all.v2023.2.Hs.symbols.gmt[Curated] (c2 gene set) from MSigDB to serve as the reference gene set for subsequent analysis, which included 66 gene sets associated with the TCA cycle, glycolysis, and fatty acid metabolism.

Based on overall survival, 131 TCGA-LAML patients were grouped into long-term survival group (OS ≥ 12 months) and short-term survival group (OS < 12 months). Firstly, GSEA was performed on long-term and short-term survival groups in the TCGA cohort based on the c2 gene set to investigate the distinct features of metabolic processes associated with survival. Secondly, we derived all 66 gene sets related to the TCA cycle, glycolysis, and fatty acid metabolism pathways from MSigDB, and performed GSEA on metabolism ^high^ and metabolism ^low^ groups based on these 66 metabolic-related gene sets. Thirdly, we conducted leading-edge analysis in GSEA software, aiming to identify the leading-edge genes. The leading-edge genes are the main drivers of the enrichment signal in enriched gene sets with FDR < 0.1 and normalized enriched score (|NES|) > 1.5 after GSEA on metabolism ^high^ and metabolism ^low^ groups.

### 2.4. Establishment and Validation of Prognostic Model

Firstly, univariate Cox regression analysis was applied on all leading-edge genes from GSEA to assess the impact of the expression level of each leading-edge gene on OS, which was accomplished by survival package in R [20]. Genes with *p*-values less than 0.05 were selected as prognosis-related genes. The LASSO regression is a statistical technique for linear regression that selects important features and prevents overfitting by shrinking some coefficients to zero. Ten-fold cross-validation is a technique used to evaluate and compare models. The LASSO regression was performed on prognosis-related genes, through glmnet packages (version 4.1.8) [21] in R, and the lambda value was selected with the smallest likelihood bias as the optimal lambda value by ten-fold cross-validation. Finally, we identify the optimal set of genes and the corresponding regression coefficients of these genes. The prognosis risk score was established with the following formula:

$$Riskscore=\sum_{i=1}^{n}\beta_{i}E_{i}$$

where *β_i_* represented the coefficient of gene*_i_* from regression results and *E_i_* represented the expression level of gene*_i_*.

The risk scores were calculated for each case in the TCGA-LAML (*n* = 131), Beat-AML (*n* = 252), and GEO cohorts (*n* = 300), based on the normalized expression data in each case. Patients were subsequently divided into high-risk and low-risk groups according to the median cutoff of the prognosis risk score. The prognostic performance was evaluated by using time-dependent receiver operating characteristic (ROC) curve analysis within three years and five years to evaluate the predictive accuracy and sensitivity of our prognostic model. The overall survival probability of AML patients in low- and high-risk groups was estimated by the Kaplan–Meier method and compared through the log rank test. A nomogram was created by rms (Regression Modeling Strategies)1 packages (version 6.3-0) [22] and survival2 packages in R (version 3.4-0).

In addition, we performed univariate and multivariate Cox regression analysis on the patients in the TCGA training cohort and the Beat-AML validation cohort to assess the validity of the metabolism-related risk score incorporated with several widely used clinical factors in predicting prognosis, which included gender, age, WBC count, bone marrow blast percentage, and cytogenetic risk.

### 2.5. Statistical Analysis

Continuous variables are presented as median and interquartile range (IQR) and compared using the Mann–Whitney U-test. Categorical variables were analyzed using Fisher’s exact test. A two-tailed *p* < 0.05 was considered statistically significant. All statistical analyses were performed using the R software 4.0.2 (The CRAN project, www.r-project.org, accessed on 1 October 2022).

### 2.6. Summary of the Methods

**Figure 1:**
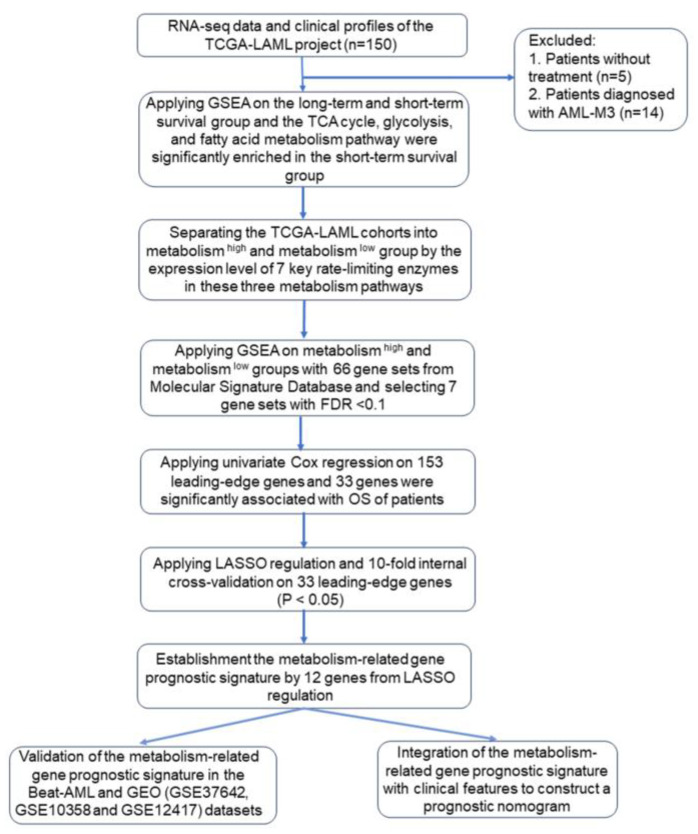
Flow chart summarizing the methods and the sequential steps performed to construct and validate the metabolism-related gene prognostic signature. TCGA-LAML, Cancer Genome Atlas-Acute Myeloid Leukemia-like; TCA cycle, tricarboxylic acid cycle; GSEA, gene set enrichment analysis; LASSO, least absolute shrinkage and selection operator.

The methods and the sequential steps performed to construct and validate the metabolism-related gene prognostic signature are presented in Figure 1.

## 3. Results

### 3.1. Comparison of the Metabolic Pathways between Long-Term and Short-Term Survival Groups in AML Patients from the TCGA-LAML Dataset

Table S2 shows the metabolic pathways that were significantly enriched (FDR < 0.25) in the gene expression data of the group with short-term survival (OS < 12 months) compared with the group with long-term survival (OS > 12 months) in the TCGA-LAML database. Among all pathways in the c2 gene set from MSigDB, the most significantly enriched metabolic pathways are associated with the TCA cycle, glycolysis, and fatty acid metabolism, which suggested these three metabolic pathways contributed the most to the impact on the survival of AML patients. Thus, we then focused on these three metabolic pathways to further explore the relationship between the metabolic signatures and survival of AML patients. By integrating the expression level of the rate-limiting enzymes in glycolysis, the TCA cycle, and fatty acid metabolism pathways, we differentiated the metabolic status of AML patients in the training cohort for the sequential study.

### 3.2. Identification of Leading-Edge Gene

After performing GSEA on the metabolism ^high^ (*n* = 65) and metabolism ^low^ (*n* = 66) group in the TCGA-LAML training cohort, we selected seven gene sets with FDR < 0.1 and |NES| > 1.5, which included the glycolysis pathway, glycolysis gluconeogenesis, citrate cycle TCA cycle, and oxidative phosphorylation pathways, glucose import, fatty acid β-oxidation, and fatty acid β-oxidation using acyl CoA oxidase pathways. Then, we identified 153 leading-edge genes with core enrichment by leading-edge analysis on the seven enriched gene sets. Table S3 shows the metabolic pathways significantly enriched in the gene expression data of the metabolism ^high^ compared with metabolism ^low^ group and the description of genes in the correspondent gene sets.

### 3.3. Establishment of Metabolism-Related Gene Prognostic Signature from Metabolism-Related Genes Associated with OS in the TCGA-LAML Dataset

Firstly, among all 153 leading-edge genes, a total of 33 leading-edge genes (*ACOXL*, *CRAT*, *SESN2*, *ABCD1*, *HSD17B10*, *ECH1*, *ECHS1*, *ETFB*, *ABCB11*, *SORT1*, *C1QTNF12*, *PEA15*, *SLC27A4*, *INSR*, *PC*, *IDH3G*, *IDH3B*, *SDHB*, *ACO2*, *SUCLG1*, *PGM1*, *HK1*, *ALDOC*, *ALDH2*, *PFKL*, *ENO1*, *PFKP*, *AKR1A1*, *CYC1*, *SDHA*, *NUP210*, *PPP2R1A*, and *HK2*) were significantly associated with the OS of AML patients (*p* < 0.05) by univariate Cox regression analysis. The full names of the 33 leading-edge genes and the metabolic pathways they belong to are summarized in Table S4.

Secondly, to further screen the most predictive genes in these 33 genes, the statistical method LASSO and ten-fold internal cross-validation were utilized. Ultimately, we determined the best lambda value (0.0625) and used the β coefficients of 12 genes (*SESN2*, *ABCB11*, *SORT1*, *SLC27A4*, *INSR*, *PC*, *SDHB*, *ALDH2*, *ENO1*, *SDHA*, *NUP210*, and *HK2*) selected by the LASSO model.

The metabolism-related risk score model was established as follows:

Risk Score = [0.0975 × log (expression level of *SESN2*)] + [0.0105 × log (expression level of *ABCB11*)] + [0.0178 × log (expression level of *SORT1*)] + [0.1655 × log (expression level of *SLC27A4*)] − [0.5768×log (expression level of *INSR*)] + [0.0089 × log (expression level of *PC*)] + [0.1712 × log (expression level of *SDHB*)] + [0.1514 × log (expression level of *ALDH2*)] + [0.3138 × log (expression level of *ENO1*)] + [0.4829 × log (expression level of *SDHA*)] + [0.2410 × log (expression level of *NUP210*)] − [0.3275 × log (expression level of *HK2*)]

**Figure 2:**
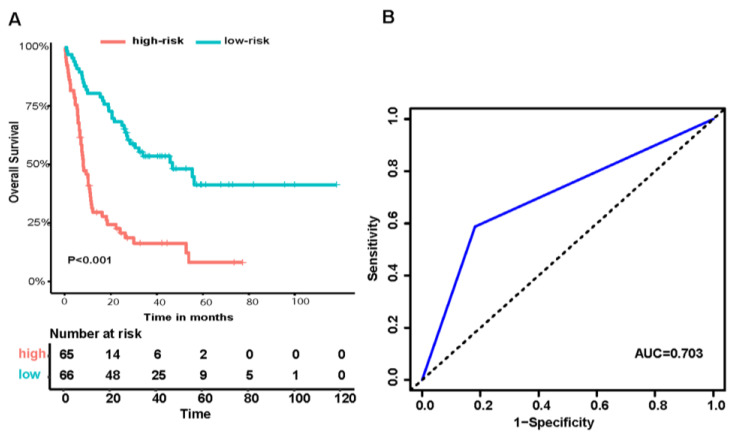
Construction of the metabolism-related gene prognostic signature in the TCGA-LAML cohort. Kaplan–Meier curves for overall survival in the two risk groups based on the prognostic signature; (**A**) ROC analysis and AUC for five-year survival of the metabolism-related risk model (**B**). The blue line shows the ROC curve.

Finally, with the median metabolism-related risk score as the cutoff value, the training set (TCGA-LAML) was separated into two groups (high-risk, *n* = 65 vs. low-risk, *n* = 66). The five-year OS of the high-risk and low-risk groups were 8.2% (95% CI, 2.6–25.7%) and 41.3% (95 CI, 29.2–58.3%, *p* <0.001, Figure 2A), respectively, and the results witnessed a survival advantage in the low-risk group. The AUC value of the metabolism-related risk for five-year OS for AML patients in the training set was 0.703 (Figure 2B), indicating that the metabolism-related gene signature had an accurate predictive capacity for prognosis in AML.

### 3.4. External Validation of Metabolism-Related Gene Prognostic Signature in GEO AML (GSE37642, GSE10358, and GSE12417) and the Beat-AML Datasets

**Figure 3:**
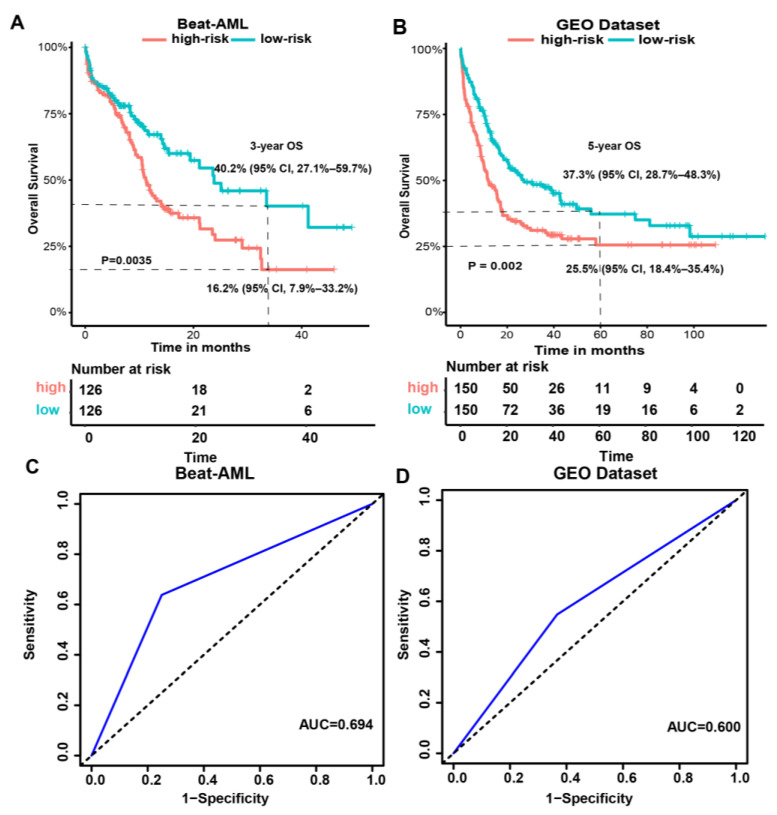
Validation of the metabolism-related gene prognostic signature in the validation datasets. Kaplan–Meier curves for overall survival based on the prognostic signature in the Beat-AML (**A**) and combined GEO cohort (**B**), ROC analysis and AUC for three-year survival of the metabolism-related risk model in the Beat-AML cohort (**C**). ROC analysis and AUC for five-year survival of the metabolism-related risk model in the combined GEO cohort (**D**). The blue lines show the ROC curves.

Both validation datasets were split into high-risk (Beat-AML, *n* = 126; GEO, *n* = 150) and low-risk groups (Beat-AML, *n* = 126; GEO, *n* = 150) by the median of the metabolism-related risk score. Figure 3A and 3B demonstrate the Kaplan–Meier curves for overall survival based on the prognostic signature in the Beat-AML and combined GEO cohort. The survival analysis indicated that, of the two validation cohorts, the samples in the high-risk groups both had significantly poorer outcomes than those in the low-risk groups (*p* = 0.0035, *p* = 0.002, respectively).

The AUC value of the metabolism-related risk model for three-year OS for AML patients in the Beat-AML cohort was 0.694 (Figure 3C), and for five-year OS in the combined GEO validation cohort was 0.600 (Figure 3D), indicating that the metabolism-related risk model was a reliable prognostic signature.

### 3.5. Metabolism-Related Gene Prognostic Signature Is an Independent Prognostic Factor

Table 3 shows the results of univariate and multivariate Cox regression analysis based on the OS in the TCGA-LAML and Beat-AML cohorts.

The univariate analysis indicated that age, WBC count, cytogenetic risk, and the metabolism-related risk score were the significant unfavorable factors associated with OS. Then, the above four factors were further included in the multivariate analysis and the metabolism-related risk score, presenting an independent prognostic factor after adjusting for other clinical variables.

**Figure 4:**
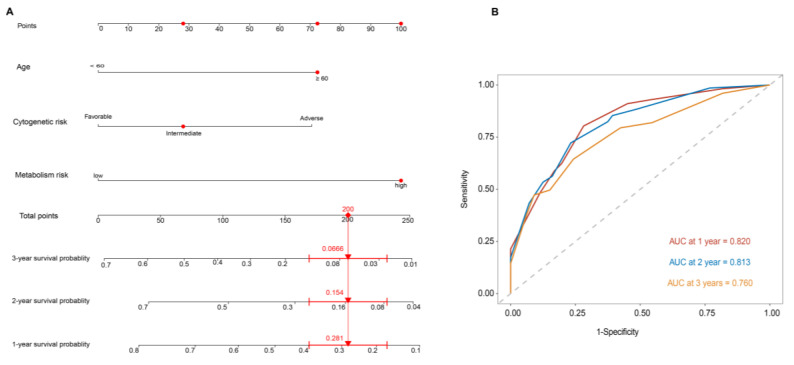
Construction of the OS-predictive nomogram for clinical application. Each line of the prognostic nomogram consists of the name of each predictive factor, including metabolism risk, age, and cytogenetic risk, on the left, and the corresponding scales lines on the right. The scales on the lines represent the factor’s range of values, and the length of the line reflects the contribution of the factor to the clinical outcome events. The scores in the nomogram, including the individual score (Points), correspond to each variable at different values. The total score (Total Points) is obtained by adding the individual scores of all variable values. According to the total score, the corresponding 1-year, 2-year, and 3-year survival probability of an individual patient could be obtained (**A**). ROC analysis and AUC for 1-year, 2-year, and 3-year survival of the nomogram in the Beat-AML cohort (**B**). For example, a 60-year-old patient with AML having an intermediate cytogenetic risk has a high metabolism risk. The individual score of cytogenetic risk, age, and metabolism risk is shown successively in the “Points” line (the red point on the line). The total score of the patient by adding three individual scores is marked in the “Total Points” line (the red point on the line). The corresponding 1-year, 2-year, and 3-year survival probability of the patient could be obtained by the corresponding straight line (the red straight line).

The construction of the OS-predictive nomogram for clinical application is demonstrated in Figure 4. After multivariate Cox proportional hazard regression, age, cytogenetic risk, and the metabolism risk score were integrated to construct a prognostic nomogram for better evaluating an individual’s risk in the clinical setting. The AUC values of the prognostic nomogram for 1-year, 2-year, and 3-year OS were 0.820, 0.813, and 0.760, respectively, indicating the favorable capability of the nomogram to estimate survival for AML patients (Figure 4B).

**Figure 5:**
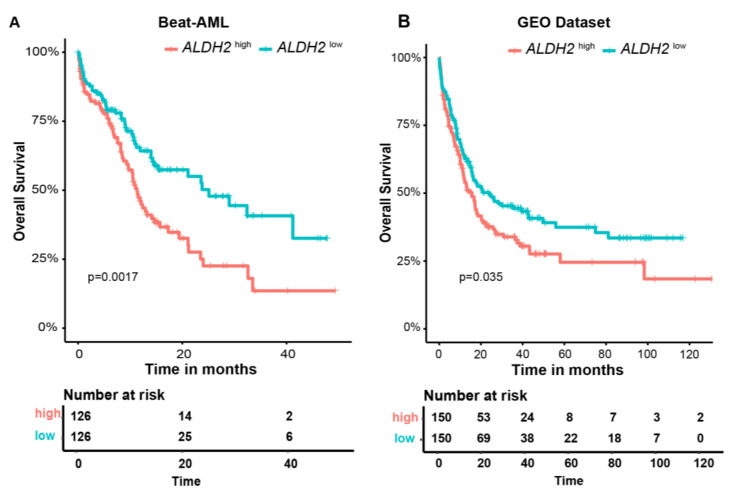
Kaplan–Meier curves for overall survival between *ALDH2* ^high^ and *ALDH2* ^low^ groups in the Beat-AML (**A**) and combined GEO cohorts (**B**).

In addition, among 12 involved metabolism-related genes, Kaplan–Meier curves showed that a high expression of *ALHD2* was significantly associated with poorer outcomes in AML patients in two validation cohorts (*p* = 0.0017, *p* = 0.035, respectively), which might be an independent biomarker of poor prognosis (Figure 5A,B). Kaplan–Meier curves for the overall survival of AML patients with high and low expression of *ABCB11*, *ENO1*, *HK2*, *INSR*, *NUP210*, *PC*, *SDHA*, *SDHB*, *SESN2*, *SLC27A4,* and *SORT1* in the Beat-AML and combined GEO cohorts are presented in Figure S1.

## 4. Discussion

AML has been studied thoroughly in the aspects of epigenomic and genomic sequencing, gene transcription, and protein expression. ELN risk stratification, the widely used prognostic system based on cytogenetics and genomics, failed to predict the accurate survival situation between heterogeneous intermediate-risk groups in AML. Previous studies demonstrated that metabolism reprogramming, including glycolysis, fatty acid metabolism, and the TCA cycle, was associated with leukemogenesis, therapeutic resistance, and poorer outcomes in AML [8,23]. Hence, we constructed a comprehensive prognostic model based on glycolysis, fatty acid metabolism, and the TCA cycle, which was an independent prognostic factor and showed a robust predictive ability in the long-term survival of AML in the training and validation sets. We separated the TCGA-LAML cohort into the high-risk and low-risk group with the median metabolism-related risk score. The results of the survival analysis witnessed a survival advantage in the low-risk group. As for the validation cohorts, a survival advantage was also demonstrated in the low-risk group in the Beat-AML and combined GEO cohort. The AUCs calculated for the two validation cohorts further verified the robustness of the metabolism-related risk signature. Our study suggested that the metabolism-related risk score could be a supplemental tool for the risk stratification of AML.

Several groups have demonstrated different metabolism signatures, focused on a specific metabolism pathway analysis, with a predictive performance of AML survival and treatment guidance. Table 4 shows a comparison of four previous studies that created a metabolism-related prognostic signature, including the study design and methods, the training and validation cohorts, the main results, the metabolism-related signatures generated by these studies, and the prognostic significance of these signatures with our study. A Chinese group generated a prognosis risk score with a panel of six serum glucose metabolism markers, which displayed an independent prognostic value in cytogenetically normal AML patients [3]. Moreover, the consistency and accuracy of a carbohydrate-metabolism-related gene prognostic signature on the predictive performance of AML survival was validated using GEO cohorts and the authors’ own cohort [24]. Another group recently developed a distinct six-lipid-metabolism-related-gene prognostic risk signature for AML [25]. Wei et al. proposed a metabolism-related prognostic signature index consisting of three metabolism-related gene pairs [26]. The combination of MRPSI and age as a composite metabolism–clinical prognostic model index demonstrated better prognostic accuracy.

As shown in Table 4, each study has a different focus, and several single metabolomic pathway-based signatures have been reported to be capable of aiding in improving the prediction accuracy of AML. However, a comprehensive metabolic signature for AML is still lacking. After performing GSEA on the short-term and long-term survivors, diverse metabolic pathways mainly including the TCA cycle, glycolysis, and fatty acid metabolism pathway were significantly enriched in the short-term survival group. Thus, our study constructed a comprehensive metabolic signature mainly focused on a combination of the TCA cycle, glycolysis, and fatty acid metabolism pathway for AML. We integrated the rates of these three metabolic pathways to differentiate the metabolism ^high/low^ group in the training cohorts and identified the leading-edge genes between the two groups to further establish the metabolism-related risk signature.

Our study created a risk model consisting of 12 metabolism-related genes, many of which have been previously implicated in the pathogenesis, progression, and prognosis of leukemia. The functions and effects of these twelve genes are shown in Table 5.

*ENO1* [27,28,29] and *PC* [31] were found to be overexpressed in several types of AML. A high expression of *SDHA* [41] and *NUP210* [33,34] were positively associated with the unfavorable prognosis of AML patients and an elevated expression of *SLC27A4* was linked to poorer clinical outcomes in several cancer types [35]. An upregulated level of *SORT1* [37,38] and downregulated *INSR* [39] were both reported in chemo-resistant or relapsed AML samples. *SDHB* [40] and *ABCB11* [43,44] were reported to play an important role in imatinib resistance. *HK2* overexpression was demonstrated to result in the chemoresistance of LSCs to DNA-damaging agents [45].

*ALDH2* is necessary for protecting hematopoietic stem and progenitor cells (HSPCs) against acetaldehyde toxicity [46]. *ALDH2* is also reported to play an important role in the chemoresistance of AML cells. The overexpression of *ALDH2* significantly increased the proliferation rate and the ability to form colonies in leukemia cell lines, resulting in increased resistance to doxorubicin [47]. The inhibition of *ALDH2* with daidzin and CVT-10216 significantly inhibited mesenchymal stromal cell (MSC)-induced ALDH activity in AML cells and sensitized them to chemotherapy [48]. Moreover, the expression levels of ALDH2 are increased in primary AML cells from elderly patients [50]. Consistent with previous research, a high expression of *ALDH2* was significantly associated with poorer outcomes of AML patients in our study, which might be related to the chemoresistance resulting from the high expression level.

There is still an urgent need for novel therapies in AML since many patients relapse. And, notably, our study highlighted the potential importance of *ALDH2* as an independent therapeutic target. Moreover, metabolism-targeted therapy has been shown to overcome chemotherapy resistance to a certain extent. The glycolytic inhibitor 2-DG combined with Ara-C could enhance cytotoxic effects in primary blast cells [3]. Statins combined with chemotherapy improved the complete remission and complete remission with incomplete count recovery rates in R/R AML [9]. Thus, metabolism-related drugs could potentially be added to chemotherapy in relapsed or refractory patients with a high-risk metabolism-related gene prognostic signature to increase their sensitivity to chemotherapy. Our study provided an effective metabolism-related prognostic signature for clinical application; high-risk patients could attempt to add inhibitors that target glycolysis, fatty acid metabolism, and the TCA cycle pathways, in addition to traditional chemotherapy.

In conclusion, we identified a novel 12-gene metabolism-related prognostic gene signature for AML by Cox regression analysis and LASSO. This gene signature could be a powerful supplemental tool for the risk stratification and outcome prediction of AML. The gene signature might play an important role in the better understanding of metabolic pathways as the potential prognostic biomarkers and therapeutic targets for AML. This metabolism-related gene prognostic signature is primarily based on bioinformatics data analysis extracted from the public database and needs to be verified in larger-scale clinical cohorts. The metabolism risk score could be utilized together with the currently known genetic alterations used for risk stratification to improve the prognostic value and develop novel treatment options to improve final outcomes.

## Figures and Tables

**Table 1:** Overview of biological and clinical data referring to patients in the TCGA-LAML, Beat-AML, and GEO datasets.

	TCGA-LAML	Beat-AML	GEO (GSE37642, GSE10358 and GSE12417)	*p*-Value
Number	131	252	300	
Age (years), median (IQR)	57.0 (43.0, 66.5)	61.0 (44.0, 71.0)	59.0 (45.0,67.0)	0.197
Gender (%)				0.880
Male	72 (55.0)	135 (53.6)	NA	
Female	59 (45.0)	117 (46.4)	NA	
WBC count at diagnosis, ×10^9^/L, median (IQR)	19.6 (5.5, 47.8)	23.0 (8.1, 56.8)	19.9 (5.1,62.7)	0.316
BM blast, %, median (IQR)	71.0 (52.0, 83.0)	72.0 (43.5, 89.0)	70.0 (53.5, 86.0)	0.959
FAB, n (%)				<0.001
M0	15 (11.5)	4 (5.2)	17 (5.7)	
M1	36 (27.5)	7 (9.1)	73 (24.4)	
M2	35 (26.7)	6 (7.8)	103 (34.4)	
M4	28 (21.4)	23 (29.9)	44 (14.7)	
M5	14 (10.7)	27 (35.1)	37 (12.4)	
M6	2 (1.5)	0 (0.0)	10 (3.3)	
M7	1 (0.8)	2 (2.6)	2 (0.7)	
Cytogenetics, n (%)				0.213
Normal	63 (48.5)	121 (50.4)	NA	
+8	6 (4.6)	10 (4.2)	NA	
del(5)	1 (0.8)	4 (1.7)	NA	
del(7)	4 (3.1)	8 (3.3)	NA	
*MLL* rearrangement	8 (6.2)	18 (7.5)	NA	
inv(16)	10 (7.7)	17 (7.1)	NA	
t(8;21)	7 (5.4)	9 (3.8)	NA	
complex	21 (16.2)	22 (9.2)	NA	
Cytogenetic risk, n (%)			<0.001	
Adverse	34 (26.4)	88 (34.9)	NA	
Favorable	17 (13.2)	85 (33.7)	NA	
Intermediate	78 (60.5)	79 (31.3)		
Follow-up duration, months, median (IQR)	18.1 (6.8, 35.7)	8.6 (4.3, 15.3)	14.4 (5.7, 37.4)	<0.001
OS events, n (%)				0.001
Dead	88 (67.2)	122 (48.4)	185 (61.7)	
Alive	43 (32.8)	130 (51.6)	115 (38.3)	

IQR, interquartile range; NA, not available; WBC, white blood cell; BM, bone marrow; OS, overall survival.

**Table 2:** The key rate-limiting enzymes in glycolysis, the TCA cycle, and fatty acid metabolism, and their corresponding gene symbols.

Metabolic Pathways	Key Rate-Limiting Enzymes	Gene Symbols
Glycolysis	Hexokinase	*HK1*, *HK2*, *HK3*
	Phosphofructokinase-1	*PFKL*, *PFKM*, *PFKP*
	Pyruvate kinase	*PKLR*, *PKM*
TCA cycle	Citrate synthase	*CS*
	Isocitrate dehydrogenase	*IDH1*, *IDH2*, *IDH3A*, *IDH3B*, *IDH3G*
	Oxoglutarate dehydrogenase complex	*OGDH*, *DLST*, *DLD*
Fatty acid metabolism	Carnitine palmitoyltransferase I	*CPT1A*, *CPT1B*, *CPT1C*

**Table 3:** Univariate and multivariate Cox regression analysis based on OS in the TCGA-LAML and Beat-AML cohorts.

	Univariable		Multivariable	
	HR (95% CI)	*p*-Value	HR (95% CI)	*p*-Value
TCGA-LAML (Training cohort)				
Gender (female vs. male)	1.03 (0.68–1.58)	0.874	\	\
Age (≥60 vs. <60)	2.38 (1.56–3.64)	<0.001	2.23 (1.42–3.52)	0.001
WBC count (≥100 vs. <100 × 10^9^/L)	2.08 (1.04–4.16)	0.039	1.75 (0.82–3.75)	0.149
Bone marrow blast (≥70% vs. <70%)	1.36 (0.89–2.07)	0.157	\	\
Cytogenetic risk *	2.88 (1.24–6.69)	0.014	1.28 (0.51–3.18)	0.600
Metabolism risk score (high vs. low)	3.39 (2.18–5.29)	<0.001	2.69 (1.66–4.35)	<0.001
Beat-AML (Validation cohort 1)				
Gender (female vs. male)	1.41 (0.98–2.03)	0.065	\	\
Age (≥60 vs. <60)	2.47 (1.36–3.62)	<0.001	2.13 (1.45–3.14)	0.001
WBC count (≥100 vs. <100 ×10^9^/L)	1.76 (0.94–3.32)	0.080	\	\
Bone marrow blast (≥70% vs. <70%)	1.15 (0.76–1.76)	0.508	\	\
Cytogenetic risk	2.71 (1.63–4.53)	<0.001	1.78 (1.02–3.10)	0.041
Metabolism risk score (high vs. low)	1.72 (1.19–2.49)	0.004	1.74 (1.13–2.68)	0.032

HR, hazard ratio; Cytogenetic risk *, referred to 2022 ELN risk classification by genetics.

**Table 4:** Comparison of all prior metabolism-related prognostic signatures for AML with our study.

Metabolism Signature	Enrolled Markers	Training Dataset	Validation Datasets	What Was Studied	Methods	Significance	Results Related to the Signature	Reference
A panel of serum glucose metabolism markers	Lactate, 2-oxoglutarate, pyruvate, 2-HG, glycerol-3-phosphate, and citrate	229 de novo AML patients enrolled in 2007 to 2010 from Rui Jin Hospital in Shanghai	171 newly diagnosed AML patients enrolled in 2011 to 2012 from 6 hematology centers	Metabolomic profiles of all serum samples, focused on the glucose metabolism	A predictive principal component analysis model	Provided strong evidence for the use of serum metabolites and metabolic pathways as novel prognostic markers and potential therapeutic targets for AML	--	Chen et al., 2014 [3]
A carbohydrate-metabolism-related gene signature	*PFKL*, *IDH3G*, *G6PD*, *DCXR*, *CYB5R3*, *CYB5R4*, *ACADS*, *MLYCD*, *PIK3CA*, and *CDIPT*	117 AML samples from the TCGA cohort	GSE37642 (*n* = 140), GSE37642 (*n* = 422), and 106 de novo AML patients in Affiliated Hospital of Southwest Medical University from January 2019 to June 2022	355 carbohydrate-metabolism-related genes were derived from the Kyoto Encyclopedia of Genes and Genomes pathway database	LASSO analysis and a multivariate Cox regression	The carbohydrate metabolism related signature was reliable and may provide theoretical support for AML prognostic judgment and treatment	*RUNX1, IDH2, WT1*, and *KRAS* mutations were more frequently in the low-risk group, and *TP53, KIT,* and *TTN* mutations were more common in the high-risk group	Yang et al., 2022 [24]
A lipid-metabolism-related gene signature	*LDLRAP1*, *PNPLA6*, *DGKA*, *PLA2G4A*, *CBR1*, and *EBP*	144 AML patients are extracted from UCSC Xena Browser	GSE71014, GSE12417, and GSE37642	26 lipid-metabolism-related pathways including 1045 genes extracted from the MSigDB	Survival analysis and then LASSO analysis	Contributed to better understanding of the use of metabolites and metabolic pathways as the potential prognostic biomarkers and therapeutic targets for AML	The common immune checkpoints were significantly upregulated in the high-risk group, indicating an immunosuppressive TME of bone marrow in the high-risk group	Li et al., 2022 [25]
A metabolism-related prognostic signature index consisting of gene pairs	*FADS1| NEU1*, *SLC2A5*| *TBXAS1*, *FADS1| PDE4B*	151 AML patients from the TCGA cohort	162 AML patients from the GSE12417 cohort and 417 AML patients from the GSE37642 cohort	Metabolism-related genes that are differentially expressed between TCGA cohort and normal bone marrow	A pairwise comparison, a univariate Cox regression, and LASSO analysis	Provided a composite metabolism and clinical model as a novel prognostic stratification method and identified several potential therapeutic drugs for AML	--	Wei et al., 2022 [26]
A metabolism-related prognostic model	12 genes shown in Table 5	131 AML patients from the TCGA-LAML cohort	252 patients from the Beat-AML cohort and 300 from GEO cohort (GSE37642, GSE10358, and GSE12417)	Genes related to glycolysis, fatty acid metabolism, and the TCA cycle pathways	A univariate Cox regression, and then LASSO analysis	Contributed to better use of metabolism reprogramming factors as prognostic marker and provide novel insights into potential metabolism target for AML treatment	--	Our study

TCGA, The Cancer Genome Atlas; LASSO, least absolute shrinkage and selection operator; TME, tumor microenvironment; UCSC, University of California, Santa Cruz; MSigDB, Molecular Signatures Database.

**Table 5:** The 12 metabolism-related genes included in the gene prognostic signature.

Genes	Full Name	Function	Effects	References
*ENO1*	α-Enolase 1	Glycolytic enzyme	Overexpressed in several types of AML	[27,28,29]
*PC*	Pyruvate carboxylase	Catalyzing the ATP-dependent carboxylation of pyruvate to oxaloacetate	Highly expressed in AML K562 cell line	[30,31]
*NUP210*	Nucleoporin 210	Involved in nucleocytoplasmic transport	Overexpressed in LSCs of pediatric AML	[32,33,34]
*SLC27A4*	Solute carrier family 27 member 4	Fatty acid transporter protein	High expression associated with poorer clinical outcomes in several cancer types	[35]
*SORT1*	Sortilin 1	Regulating lipoprotein metabolism	Significantly associated with relapse and/or B-ALL-related death and upregulated in chemo-resistant AML samples	[36,37,38]
*INSR*	Insulin receptor	Binding of insulin to activate the insulin signaling pathway	Downregulated as a predictive gene for relapse among AML	[39]
*SDHB*	Succinate dehydrogenase complex iron sulfur subunit B	Encoding the iron–sulfur protein subunit of the succinate dehydrogenase enzyme complex, a complex of the mitochondrial respiratory chain	Decreased in imatinib-resistant BCR-ABL1 cells. SDHB mutations in leukemic T cells involved in cellular pre-adaptation to hypoxia.	[40]
*SDHA*	Succinate dehydrogenase complex flavoprotein subunit A	Encoding a major catalytic subunit of dehydrogenase enzyme complex, a complex of the mitochondrial respiratory chain	Significantly associated with poor survival of leukemia patients. Inhibition of SDHA with venetoclax and azacitidine led to LSC death	[41]
*ABCB11*	ATP binding cassette subfamily B member 11	The major canalicular bile salt export pump	Significantly associated with the achievement of major molecular response with first-line imatinib treatment	[42,43,44]
*HK2*	Hexokinase 2	Phosphorylating glucose to produce glucose-6-phosphate	Overexpression resulted in chemoresistance of LSCs to DNA-damaging agents	[45]
*ALDH2*	Aldehyde dehydrogenase 2	Oxidizing aldehydes	Overexpression in leukemia cell lines resulted in increased resistance to doxorubicin	[46,47,48]
SESN2	Sestrin 2	Catalyzing the reduction of hyperoxidized peroxiredoxins	Knockdown in T-ALL cells decreased the rate of mitochondrial respiration	[49]

## Data Availability

The data presented in this study are openly available in the TCGA-LAML database at https://gdc.cancer.gov/about-data/publications/laml_2012 [10], GSE37642 database at https://www.ncbi.nlm.nih.gov/geo/query/acc.cgi?acc=GSE37642 [11], GSE10358 database at https://www.ncbi.nlm.nih.gov/geo/query/acc.cgi?acc=GSE10358 [12], GSE12417 database at https://www.ncbi.nlm.nih.gov/geo/query/acc.cgi?acc=GSE12417 [13], and the Beat-AML database at https://www.cancer.gov/ccg/blog/2019/beataml [14].

## Supplementary Materials

The following supporting information can be downloaded at: https://www.mdpi.com/article/10.3390/genes15010063/s1, Figure S1: Kaplan–Meier curves for overall survival of AML patients with high and low expression of *ABCB11*, *ENO1*, *HK2*, *INSR*, *NUP210*, *PC*, *SDHA*, *SDHB*, *SESN2*, *SLC27A4* and *SORT1* in the Beat-AML and GEO cohorts ; Table S1: The overall features of the TCGA-LAML, Beat-AML, GSE37642, GSE10358, and GSE12417 databases; Table S2: Metabolic pathways that were significantly enriched (FDR < 0.25) in the gene expression data of the group with short-term survival (OS < 12 months) compared with the group with long-term survival (OS > 12 months) in the TCGA-LAML database; Table S3: Metabolic pathways that were significantly enriched (FDR < 0.2 and |NES| > 1.5) in the gene expression data of the metabolism ^high^ compared with metabolism ^low^ group in the TCGA-LAML; Table S4: The metabolism pathways of 33 prognosis-related genes identified by univariate Cox regression analysis.
