# Supplementary material for: A Comprehensive Metabolism-Related Gene Signature Predicts the Survival of Patients with Acute Myeloid Leukemia
Source: Genes (Basel). 2023 Dec 31;15(1):63. doi: 10.3390/genes15010063 (PMC10815187; doi:10.3390/genes15010063)
Supplement: Supplementary file 1 [file genes-15-00063-s001.zip › genes-2739229-supplementary.pdf]

Supplementary Materials

Methods

Differentiation of metabolic status of the patients by the expression of corresponding rate-limiting enzymes

Firstly, we used the average of the sum of HK1, HK2, and HK3 expression values to represent the expression value of hexokinase. This way, the expression value of phosphofructoki-nase-1 and pyruvate kinase were calculated. Secondly, the sum of the expression values of these three rate-limiting enzymes in glycolysis was calculated to represent the value of glycolysis pathway activity. Similarly, we calculated the sum of the expression values of corresponding rate-limiting enzymes of the TCA cycle pathway and fatty acid metabolism pathway. Thirdly, we calculated the sum of the values of glycolysis, fatty acid and, the TCA cycle activity to represent the metabolism value. Finally, we separated the TCGA-LAML training cohort into metabolism ^{high} and metabolism ^{low} groups by the median metabolism value. The Formula is as follows:

$$\text{Value of Glycolysis activity} = \frac{(E_{HK1} + E_{HK} \ \ + E_{HK3})}{3} + \frac{(E_{PFKL} + E_{PFKM} + E_{PFKP})}{3} + \frac{(E_{PKLR} + E_{PKM})}{2}$$

$$\text{Value of TCA cycle activity} = E_{CS} + \frac{(E_{IDH} \ \ + E_{IDH2} + E_{IDH3A} + E_{IDH3B} + E_{IDH3G})}{5} + \frac{(E_{OGDH} + E_{DLST} + E_{DLD})}{3}$$

$$\text{Value of Fatty acid metbolism activity} = \frac{(E_{CPT1A} + E_{CPT1B} + E_{CPT} \ \ )}{3}$$

$$\text{Metbolism value} = \text{ value of Glycolysis activiy} + \text{value of TCA cycle activiy} + \text{value of Fatty acid metbolism activiy}$$

Where E is the expression value of key rate-limiting enzyme genes.

Table S1. The overall features of the TCGA-LAML, Beat-AML, GSE37642, GSE10358, and GSE12417 databases.

	TCGA-LAML	Beat-AML	GSE37642	GSE10358	GSE12417
Number of cases	131	252	91	73	136
The time frames when diagnosed	2001-2010	NA	NA	NA	NA
Created in the year	2007	2015	2012	2008	2008

NA, not available.

Table S2. Metabolic pathways that were significantly enriched (FDR<0.25) in the gene expression data of the group with short-term survival (OS<12 months) compared with the group with long-term survival (OS>12 months) in the TCGA-LAML database.

**Gene set name from Molecular Signature Database**	**Description of Gene set**	**Size [1]**	**NES [2]**	**FDR [3]**
GOBP_FRUCTOSE_1_6_BISPHOSPHATE_METABOLIC_PROCESS[4]	Genes involved in the chemical reactions and pathways involving fructose 1,6-bisphosphate collected by Gene Ontology	9	-1.76	0.246

MOOTHA_GLUCONEOGENESIS[5]	Genes involved in gluconeogenesis collected by Broad Institute	29	-1.56	0.246
MODULE_152[6]	Genes involved in metabolism in Cancer collected by Broad Institute	122	-1.55	0.228
REACTOME_PYRUVATE_METABOLISM_AND_CITRIC_ACID_TCA_CYCLE[7]	Pyruvate metabolism and TCA cycle collected by Reactome Database	54	-1.54	0.224
MOOTHA_TCA	Genes involved in TCA cycle collected by Broad Institute	16	-1.53	0.212
MODULE_307	Genes involved in metabolism in Cancer collected by Broad Institute	25	-1.53	0.203
REACTOME_MITOCHONDRIAL_FATTY_ACID_BETA_OXIDATION	Genes involved in Mitochondrial Fatty Acid Beta-Oxidation collected by Reactome Database	37	-1.52	0.197
BIOCARTA_ETC_PATHWAY[8]	Genes involved in Electron Transport Reaction in Mitochondria collected by BioCarta Database	10	-1.52	0.183
KEGG_OXIDATIVE_PHOSPHORYLATION[9]	Genes involved in Oxidative phosphorylation collected by Kyoto Encyclopedia of Genes and Genomes	125	-1.51	0.179
WP_TCA_CYCLE_AKA_KREBS_OR_CITRIC_ACID_CYCLE[10]	Genes involved in TCA cycle collected by WikiPathways Database	18	-1.50	0.180
REACTOME_CITRIC_ACID_CYCLE_TCA_CYCLE	Genes involved in TCA cycle collected by Reactome Database	22	-1.48	0.193
WP_AEROBIC_GLYCOLYSIS	Genes involved in aerobic glycolysis collected by WikiPathways Database	11	-1.48	0.185
MOOTHA_FFA_OXYDATION	Genes involved in free fatty acid oxidation collectdy by Broad Institute	23	-1.45	0.206
MOOTHA_ROS	Genes involved in Reactive oxidative species collected by Broad Institute	7	-1.43	0.218

WP_OXIDATIVE_STRESS_RESPONSE	Genes involved in oxidative stress resoibse by WikiPathways	28	-1.43	0.213
REACTOME_FATTY_ACID_METABOLISM	Genes involved in fatty acid metabolism collected by Reactome	171	-1.40	0.246

1. Size, the number of genes enriched in the gene set; 2. FDR, false discovery rate; 3. NES, normalized enriched score; 4. GOBP, Gene Ontology Biological Process; 5. MOOTHA, gene set created by Broad Institute; 6. MODULE, gene involved in cancer collected by Broad Institute; 7. REACTOMEN, Reactome Database; 8. BIOCARTA, BioCarta Database; 9. KEGG, Kyoto Encyclopedia of Genes and Genomes; 10. WP, WikiPathways database.

Table S3. Metabolic pathways that were significantly enriched (FDR<0.2 and |NES|>1.5) in the gene expression data of the metabolism ^{high} compared with metabolism ^{low} group in the TCGA-LAML.

Gene set name from MSigDB	Description of Gene set	Size[1]	NES[2]	FDR[3]
GOBP_FATTY_ACID_BETA_OXIDATION[4]	Genes involved in the fatty acid beta oxidation collected by Gene Ontology	75	1.83	0.042
GOBP_GLUCOSE_IMPORT	Genes involved in glucose import collected by Gene Ontology	63	1.76	0.042
KEGG_CITRATE_CYCLE_TCA_CYCLE[5]	Genes involved in citrate cycle TCA cycle collected by Kyoto Encyclopedia of Genes and Genomes	30	1.57	0.081
KEGG_GLYCOLYSIS_GLUCONEOGENESIS	Genes involved in Glycolysis Gluconeogenesis collected by Kyoto Encyclopedia of Genes and Genomes	54	1.61	0.080
KEGG_OXIDATIVE_PHOSPHORYLATION	Genes involved in oxidative phosphorylation collected by Kyoto Encyclopedia of Genes and Genomes	125	1.59	0.082
REACTOME_GLYCOLYSIS[6]	Genes involved in glycolysis collected by Reactome	69	2.04	0.007
REACTOME_GLUCOSE_METABOLISM	Genes involved in glucose metabolism by Reactome	87	2.06	0.007

1. Size, the number of genes enriched in the gene set; 2. FDR, false discovery rate; 3. NES, normalized enriched score; 4. GOBP, Gene Ontology Biological Process; 5. KEGG, Kyoto Encyclopedia of Genes and Genomes; 6. REACTOMEN, Reactome Database.

Table S4. The metabolism pathways of 33 prognosis-related genes identified by univariate Cox regression analysis

Gene	Full name of gene	Related Metabolism pathway
ACOXL	Acyl-CoA Oxidase Like	Fatty acid metabolism
CRAT	Carnitine O-Acetyltransferase	Fatty acid metabolism
SESN2	Sestrin 2	Fatty acid metabolism
ABCD1	ATP Binding Cassette Subfamily D Member 1	Fatty acid metabolism
HSD17B10	Hydroxysteroid 17-Beta Dehydrogenase 10	Fatty acid metabolism
ECH1	Enoyl-CoA Hydratase 1	Fatty acid metabolism
ECHS1	Enoyl-CoA Hydratase, Short Chain 1	Fatty acid metabolism
ETFB	Electron Transfer Flavoprotein Subunit Beta	Fatty acid metabolism
ABCB11	ATP Binding Cassette Subfamily B Member 11	Fatty acid metabolism
SORT1	Sortilin 1	Glycolysis
C1QTNF12	C1q And TNF Related 12	Glycolysis
PEA15	Proliferation And Apoptosis Adaptor Protein 15	Glycolysis
SLC27A4	Solute Carrier Family 27 Member 4	Glycolysis
INSR	Insulin Receptor	Glycolysis
PC	Pyruvate Carboxylase	Glycolysis
IDH3G	Isocitrate Dehydrogenase (NAD(+)) 3 Non-Catalytic Subunit Gamma	TCA cycle
IDH3B	Isocitrate Dehydrogenase (NAD(+)) 3 Non-Catalytic Subunit Beta	TCA cycle
SDHB	Succinate Dehydrogenase Complex Iron Sulfur Subunit B	TCA cycle
ACO2	Aconitase 2	TCA cycle
SUCLG1	Succinate-CoA Ligase GDP/ADP-Forming Subunit Alpha	TCA cycle
PGM1	Phosphoglucomutase 1	Glycolysis
HK1	Hexokinase 1	Glycolysis
ALDOC	Aldolase, Fructose-Bisphosphate C	Glycolysis
ALDH2	Aldehyde Dehydrogenase 2	Fatty acid metabolism
PFKL	Phosphofructokinase, Liver Type	Glycolysis
ENO1	α-Enolase 1	Glycolysis
PFKP	Phosphofructokinase, Platelet	Glycolysis

AKR1A1	Aldo-Keto Reductase Family 1 Member A1	Glycolysis
CYC1	Cytochrome C1	TCA cycle
SDHA	Succinate Dehydrogenase Complex Flavoprotein Subunit A	TCA cycle
NUP210	Nucleoporin 210	Glycolysis
PPP2R1A	Protein Phosphatase 2 Scaffold Subunit Aalpha	Glycolysis
HK2	Hexokinase 2	Glycolysis

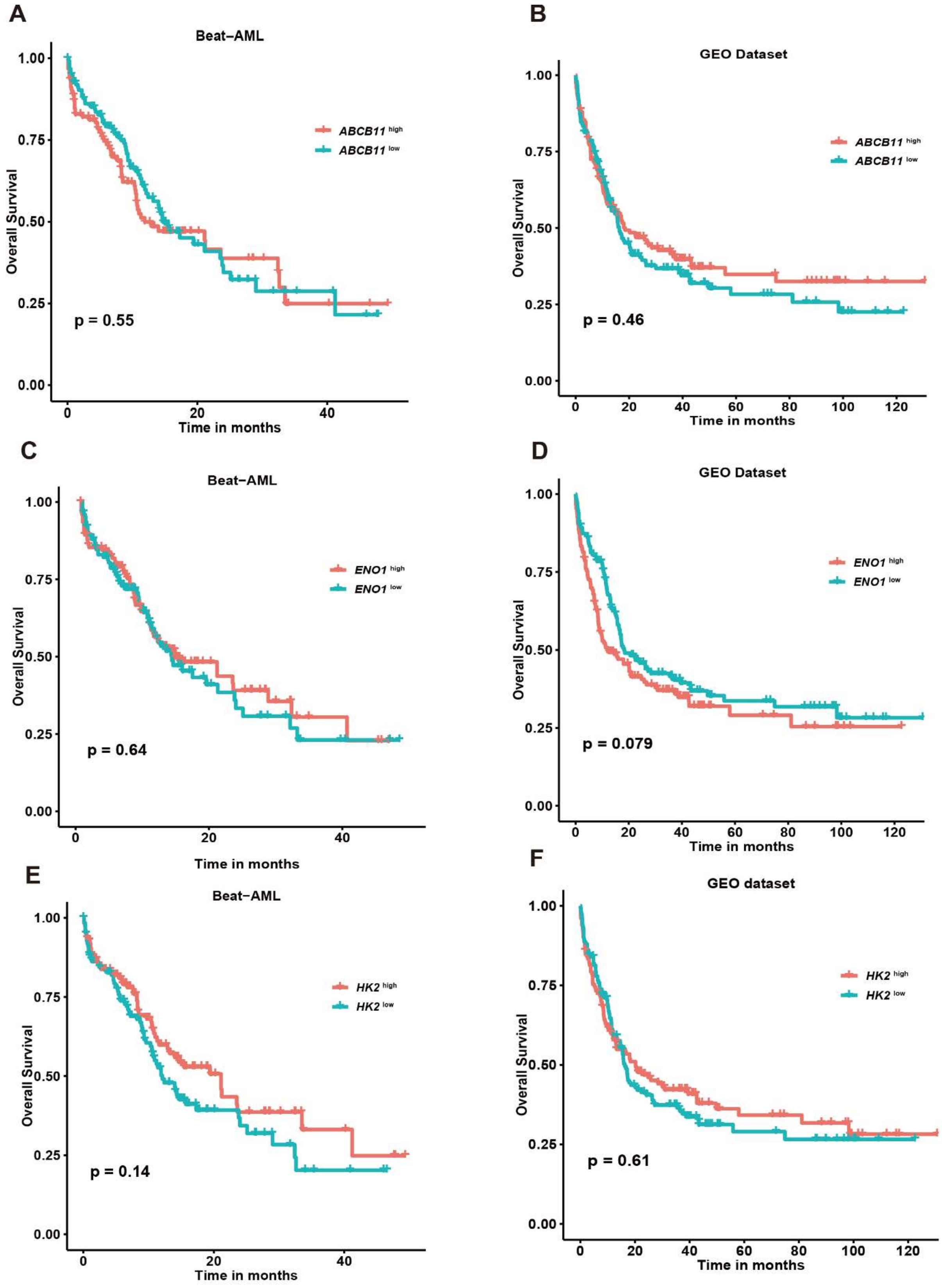
A
Beat−AML
ABCB11 high
ABCB11 low
Overall Survival
p = 0.55
Time in months
B
GEO Dataset
ABCB11 high
ABCB11 low
p = 0.46
C
Beat−AML
ENO1 high
ENO1 low
p = 0.64
D
GEO Dataset
ENO1 high
ENO1 low
p = 0.079
E
Beat−AML
HK2 high
HK2 low
p = 0.14
F
GEO dataset
HK2 high
HK2 low
p = 0.61

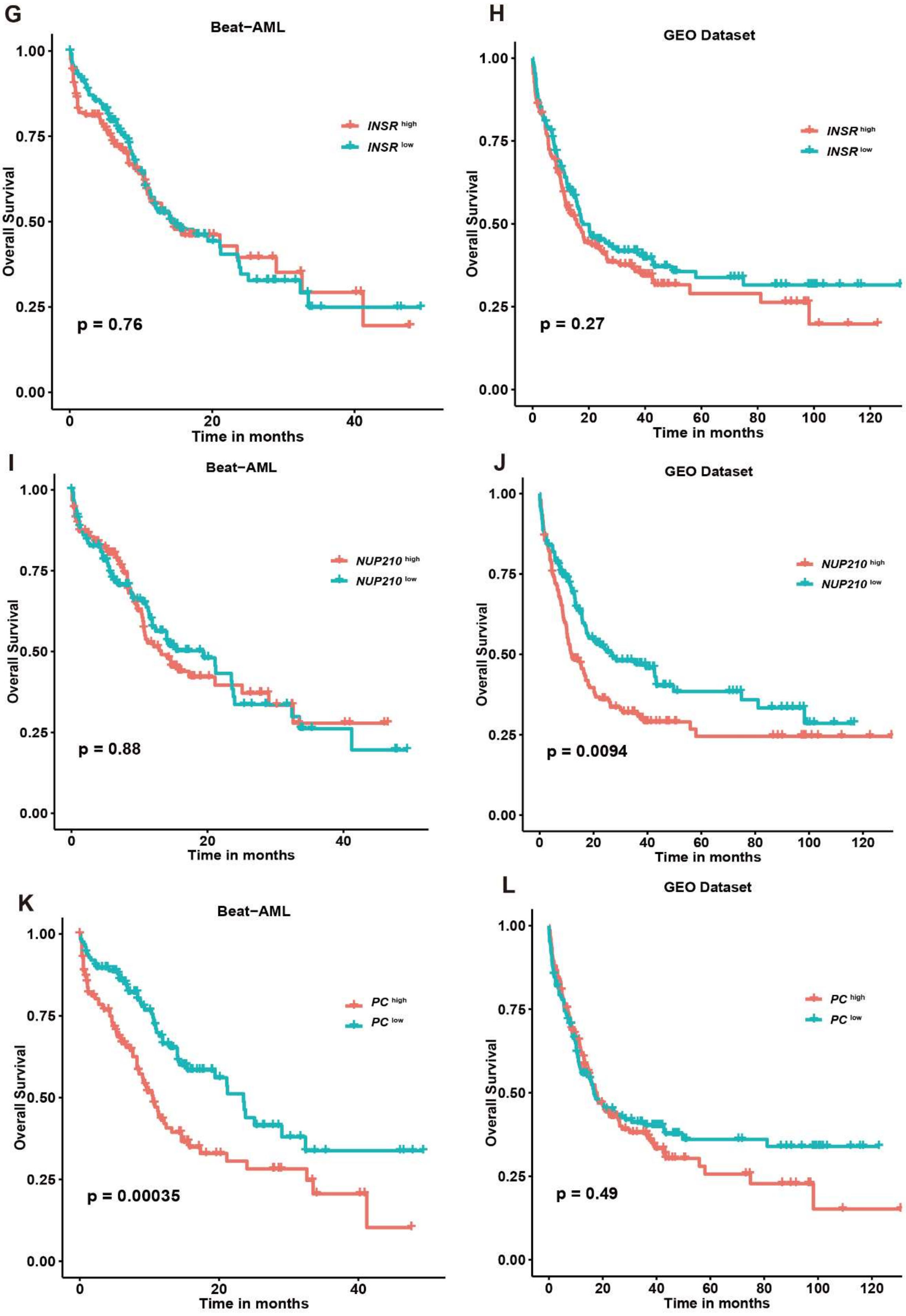
G
Beat−AML
INSR high
INSR low
p = 0.76
Overall Survival
Time in months
H
GEO Dataset
INSR high
INSR low
p = 0.27
Overall Survival
Time in months
I
Beat−AML
NUP210 high
NUP210 low
p = 0.88
Overall Survival
Time in months
J
GEO Dataset
NUP210 high
NUP210 low
p = 0.0094
Overall Survival
Time in months
K
Beat−AML
PC high
PC low
p = 0.00035
Overall Survival
Time in months
L
GEO Dataset
PC high
PC low
p = 0.49
Overall Survival
Time in months

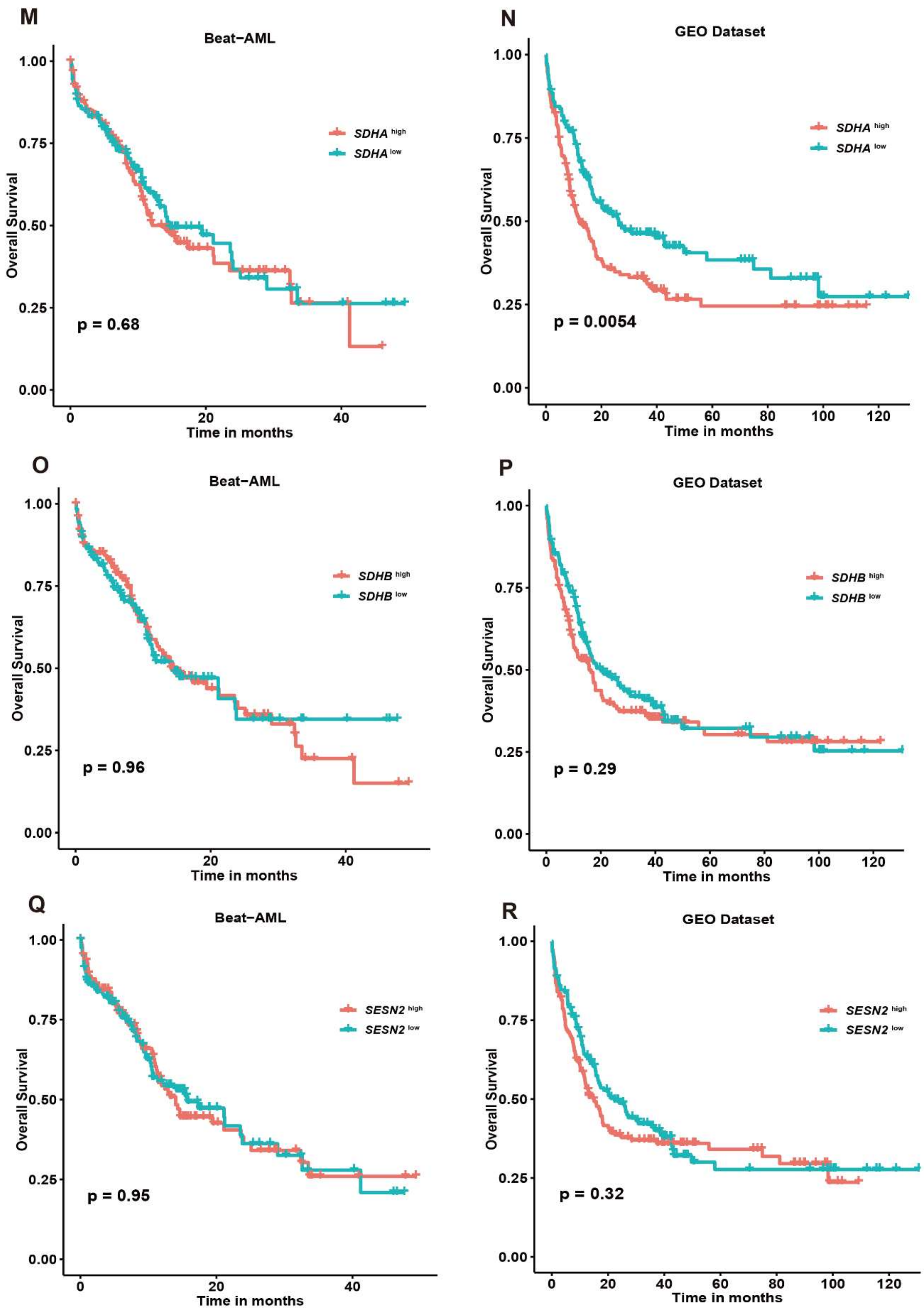
M
Beat−AML
1.00
0.75
0.50
0.25
0.00
Overall Survival
SDHA high
SDHA low
p = 0.68
0
20
40
Time in months
N
GEO Dataset
SDHA high
SDHA low
p = 0.0054
0
20
40
60
80
100
120
Time in months
O
Beat−AML
SDHB high
SDHB low
p = 0.96
P
GEO Dataset
SDHB high
SDHB low
p = 0.29
Q
Beat−AML
SESN2 high
SESN2 low
p = 0.95
R
GEO Dataset
SESN2 high
SESN2 low
p = 0.32

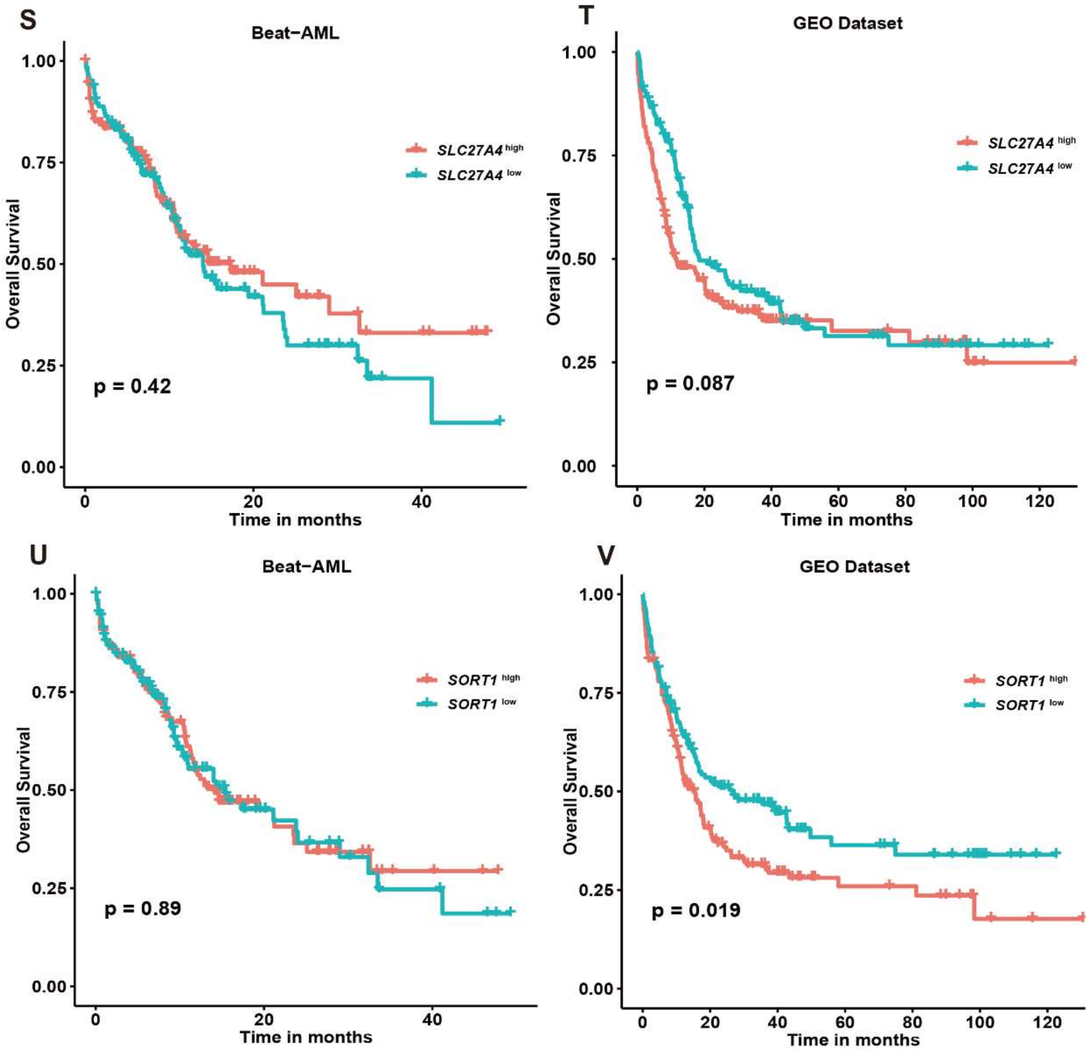

Figure S1. Kaplan–Meier curves for overall survival of AML patients with high and low expression of *ABCB11, ENO1*, *HK2*, *INSR, NUP210, PC, SDHA, SDHB, SESN2, SLC27A4* and *SORT1* in the Beat-AML (A, C, E, G, I, K, M, O, Q, S, U) and GEO cohorts (B, D, F, H, J, L, N, P, R, T, V).
